# A metabolic link between mitochondrial ATP synthesis and liver glycogen metabolism: NMR study in rats re-fed with butyrate and/or glucose

**DOI:** 10.1186/1743-7075-8-38

**Published:** 2011-06-15

**Authors:** Jean-Louis Gallis, Henri Gin, Hélène Roumes, Marie-Christine Beauvieux

**Affiliations:** 1 Centre de Résonance Magnétique des Systèmes Biologiques, UMR 5536, Université Bordeaux Segalen, CNRS, LabEx TRAIL-IBIO, 146 rue Léo Saignat, F-33076, Bordeaux Cedex, France; 2 Service de Nutrition et Diabétologie, Hôpital Haut-Lévêque, Avenue de Magellan, F-33600 Pessac, France

## Abstract

**Background:**

Butyrate, end-product of intestinal fermentation, is known to impair oxidative phosphorylation in rat liver and could disturb glycogen synthesis depending on the ATP supplied by mitochondrial oxidative phosphorylation and cytosolic glycolysis.

**Methods:**

In 48 hr-fasting rats, hepatic changes of glycogen and total ATP contents and unidirectional flux of mitochondrial ATP synthesis were evaluated by *ex vivo *^31^P NMR immediately after perfusion and isolation of liver, from 0 to 10 hours after force-feeding with (butyrate 1.90 mg + glucose 14.0 mg.g^-1 ^body weight) or isocaloric glucose (18.2 mg.g^-1 ^bw); measurements reflected *in vivo *situation at each time of liver excision. The contribution of energetic metabolism to glycogen metabolism was estimated.

**Results:**

A net linear flux of glycogen synthesis (~11.10 ± 0.60 μmol glucosyl units.h^-1^.g^-1 ^liver wet weight) occurred until the 6^th ^hr post-feeding in both groups, whereas butyrate delayed it until the 8^th ^hr. A linear correlation between total ATP and glycogen contents was obtained (r^2 ^= 0.99) only during net glycogen synthesis. Mitochondrial ATP turnover, calculated after specific inhibition of glycolysis, was stable (~0.70 ± 0.25 μmol.min^-1^.g^-1 ^liver ww) during the first two hr whatever the force-feeding, and increased transiently about two-fold at the 3^rd ^hr in glucose. Butyrate delayed the transient increase (1.80 ± 0.33 μmol.min^-1^.g^-1 ^liver ww) to the 6^th ^hr post-feeding. Net glycogenolysis always appeared after the 8^th ^hr, whereas flux of mitochondrial ATP synthesis returned to near basal level (0.91 ± 0.19 μmol.min^-1^.g^-1 ^liver ww).

**Conclusion:**

In liver from 48 hr-starved rats, the energy need for net glycogen synthesis from exogenous glucose corresponds to ~50% of basal mitochondrial ATP turnover. The evidence of a late and transient increase in mitochondrial ATP turnover reflects an energetic need, probably linked to a glycogen cycling. Butyrate, known to reduce oxidative phosphorylation yield and to induce a glucose-sparing effect, delayed the transient increase in mitochondrial ATP turnover and hence energy contribution to glycogen metabolism.

## Background

Among the short chain fatty acids (SCFA), butyrate is a natural nutrient found in food (i.e. butter and milk product) and is also produced physiologically (from 40 to 100 mmol) from intestinal fermentation of fiber [1]. Recent findings in the field of gut microbial flora strongly suggest that the symbiotic relationship between the intestinal microbiota and the human host can influence health [2]. Gut microbiota clearly affect the host nutritional metabolism with consequences for energy storage, which implies mechanistic interactions between events occurring in the colon and the regulation of energy metabolism [3].

The main site for butyrate metabolism is the liver since hepatic removal close to 100% has been evidenced in Wistar rats adapted to a high-fiber diet [4]. A recent *in vivo *study in human [5] evidenced that SCFAs were released by the gut (34.9 ± 9.1 μmol. kg^-1 ^body weight. h^-1^) in the circulatory system, while the gut butyrate release was counterbalanced by butyrate hepatic uptake (-3.8 ± 1.6 μmol. kg^-1 ^body weight. h^-1^). This indicated that the liver is highly involved in butyrate metabolism.

Fatty acids (FA) are both substrates and effectors of the hepatic oxidative pathways. They are β-oxidized in mitochondria and some of them (namely long chain fatty acids) are known to have a decoupling-like effect on mitochondrial oxidative phosphorylation [6,7]. We previously showed that the direct perfusion of isolated and perfused rat liver with short and medium chain FA (butyrate, octanoate) decreased the ATP content [8]. More recently, we demonstrated in isolated rat liver that a butyrate perfusion of the organ decreases the oxidative phosphorylation yield in the whole organ by decreasing the mitochondrial synthesis flux of ATP [9].

The liver is the main site of glycogen contribution to the regulation of blood glucose. Glycogen content varies during the day, generally increasing from a nadir at the end of the post-absorptive period to a maximal content about 4-5 hours after a meal. Glycogen synthesis, which is located in cytosol, depends on the UTP supply and hence on ATP supply resulting from both mitochondrial oxidative phosphorylation and cytosolic glycolysis. There is in isolated rat liver a linear, positive, glucose-dependent and insulin-dependent correlation between the change in ATP content and that of glycogen [10]. Glucose or glycogen cycling may also occur [11], raising the question of the contribution of ATP turnover owing to the large energy cost of the gluconeogenesis pathway. Studies have been performed with fatty acids concerning the hepatic fluxes of glucose autoregulation which could implicate a glucose or glycogen cycling [12-16]; however, to our knowledge, nothing is reported about the energetic cost of the effect of fatty acid on a potential cycling. Owing to the impairing effect of butyrate on oxidative phosphorylation, it can be hypothesized that butyrate disturbs the energy supply necessary for glycogen synthesis.

The present study sought to evaluate the effects of butyrate added to glucose feeding in rat on mitochondrial ATP synthesis in the liver and to determine whether butyrate could interact with glycogen storage, the protocol reflecting *in vivo *situation. Indeed, the originality of the present work was to monitor the changes of mitochondrial ATP turnover within a long post-feeding period, the changes reflecting integrated physiological responses of the whole liver to the kind of diet. It differed from our previous estimation of ATP turnover performed *ex vivo *in which the substrates were added to the perfusate of the isolated liver, excluding all digestive processes and regulations at the whole-body scale [8,9].

Recent nuclear magnetic resonance (NMR) developments made it possible to study hepatocellular rates of global ATP synthesis *in vivo *by using magnetization saturation transfer method [17] or on hepatic freeze-trapped biopsies subsequently treated for NMR analysis by cryo-NMR method [18]. Magnetization saturation transfer cannot be used to estimate the mitochondrial contribution to ATP supply [19] because the rate of liver oxidative phosphorylation is too slow to be accurately evaluated by this method [20]. Moreover, these methods do not allow discrimination between mitochondrial and glycolytic ATP production, and the chemical ischemia (specific inhibition of oxidative phosphorylation) coupled with NMR spectroscopy is the only method leading to such a discrimination [9,21].

In the present study, we monitor in glycogen-depleted liver of 48 hr-fasted rats, the glycogen recovery and the changes in mitochondrial ATP turnover induced by feeding of 48 hr-fasted rats with glucose alone or glucose plus butyrate. Hence, glycogen content, total ATP content and mitochondrial ATP supply were evaluated on the perfused and isolated liver, immediately after sacrifice.

## Methods and materials

### Chemicals

Glucose and butyrate were purchased from Sigma Chemical (St. Louis, Missouri, USA) except where otherwise specified.

### Animals

Male Wistar rats (Centre d'élevage Depré, St Doulchard, France) weighing 90-120 g were fed *ad libitum *with a balanced diet: carbohydrates (65%), proteins (16%), water (12%), minerals (5%), fibers (4%) and lipids (3%) amounting to 12.75 MJ/kg food as previously described [8] (Table 1). They were housed in hanging cages in a room with constant airflow, controlled temperature (21-23°C) and hygrometry, and a 12 hr light/dark system.

**Table 1:** Formulation of rat diet

Formula	(%)
Cereals	76.55

Vegetal proteins^1^	14.1

Animal proteins^2^	5

Vitamins and mineral mix	4.35

**Fatty acids**	**(mg/kg)**

Palmitic acid	6450

Palmitoleic acid	750

Stearic acid	4500

Oleic acid	8500

Linoleic acid	13850

Linolenic acid	90

Energy supply was 12.75 MJ/kg food, corresponding to 55.85% carbohydrates (with 39.90% starch), 3.95% fiber, 4.05% lipids, 18.70% proteins, 5.35% minerals and 12.1% water.

^1^Consisting of soybeans and yeast.

^2^Provided by fish.

### Specific force-feeding

Rats were fasted for 48 hr in order to totally deplete their hepatic glycogen store; a fasting period of 48 hr is currently used in various protocols concerning liver metabolic studies in rats [22-24]. They were then fed with an intragastric bolus of one of the following mixtures: (i) force-feeding consisting in 18.2 mg glucose/g body weight, or (ii) force-feeding consisting in 14 mg glucose +1.9 mg butyrate/g body weight calculated to be isocaloric to force-feeding with glucose alone (7.28 cal). A significant difference (-23%) was thus obtained in the energy supply of the glucidic component. All mixtures were diluted with water for a total force-feeding (FF) volume of 1.8 ml/100 g body weight, thus respecting the maximal recommendations of 20 ml/kg of body weight. Intragastric administration with a cannula (Harvard apparatus; 16 gauge diameter; 4 inches long) was performed within 1 minute. This mode of administration, which avoids the repulsion linked to the taste of butyrate, allows the consumption of dietary nutrients to be rigorously controlled [25].

The laboratory is licensed for animal experiments (French Agriculture Department). The protocol complied with 1999 UFAW guidelines [26] and was approved by the Regional Ethics Committee for Animal Experiments in our University.

### Preparation of animals for NMR measurements

**Figure 1:**
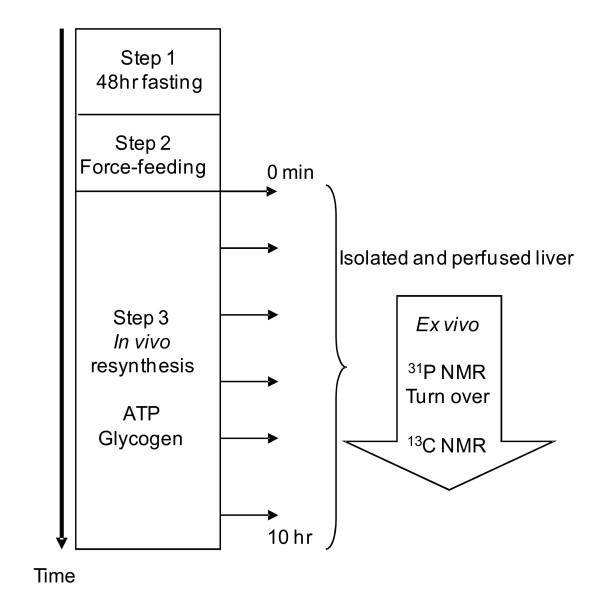
**Sequences of protocol applied to measure resynthesis of total ATP and glycogen contents, and unidirectional flux of mitochondrial ATP synthesis**. All the processes post-feeding occurred *in vivo *in integrated physiological conditions. The *ex vivo *step is the only way to estimate the mitochondrial contribution to ATP synthesis. The *ex vivo *step performed rapidly at each time post force-feeding reflects the in *vivo *situation. The total ATP and glycogen contents in controls fed *ad libitum *were 2.70 ± 0.30 and 74.97 ± 11.17 μmol/g liver wet weight, respectively. In 48-hr fasted controls, total ATP and glycogen contents were 0.95 ± 0.29 and 0.80 ± 0.10 μmol/g liver ww, respectively. In the force-feeding step, the bolus (expressed per g body weight) was isocaloric (7.28 kcal): 18.2 mg glucose or 14 mg glucose + 1.9 mg butyrate.

Animals were anaesthetized by intraperitoneal injection of pentobarbital sodium (50 mg/kg of rat) at different times from the onset to the 10^th ^hr of feeding. To avoid ischemic degradation of ATP, liver was perfused through the portal vein and then excised for NMR measurements. The number of rats (n) varied from 6 to 12 for each time point. The protocol is summarized in Figure 1.

The liver *ex vivo *antegrade perfusion technique through the portal vein was performed as essentially described by Exton [27] and then modified for NMR spectroscopy [28-30]. Briefly the liver (4-6 g) was perfused with isotonic Krebs-Henseleit buffer containing glucose (30 mmol/L) and insulin (120 mUI/L) [12,31] (glucose concentration was chosen to maintain a high level of energetic metabolism and to avoid that its concentration becomes limiting for the velocity of energetic metabolism); the perfusate was pumped through a thermostatically controlled membrane oxygenator gassed with 95% O_2 _- 5% CO_2 _(37°C), at a flow perfusion of 4 ml/min.g liver wet weight [32,9,10] in a non recirculation mode. In order to avoid interference with non-hepatic ATP and the above conditions ensuring good oxygenation, no erythrocytes were used. The perfused liver was then excised from the rat abdomen and transferred to a 20 mm-diameter NMR cell.

### NMR methodology

Determinations of total ATP and glycogen contents and of mitochondrial ATP turnover at each time point after feeding have been performed immediately after liver excision, thereby reflecting *in vivo *situation at the time of liver excision.

The spectra were obtained using a ^31^P/^13^C double-tuned 20 mm probe operating at 9.4 T. Liver ATP content was monitored by ^31^P NMR and carbohydrate content in natural abundance was assessed by ^13^C NMR. ^31^P and ^13^C NMR spectra were recorded at 161.9 and 100.6 MHz respectively on a DPX400 spectrometer (Bruker). The magnetic field was adjusted to the water proton signal. ^31^P NMR spectra were obtained without proton decoupling (each spectrum: 148 free induction decays, FIDs, 1 min 20 sec) throughout the sequence protocol. During inhibitor addition sequences, spectra were acquired every 20 sec (40 FIDs) in order to increase the determination accuracy. Radiofrequency pulses (70° flip angle) and 10,000 Hz spectral width were used for data acquisition. ^13^C NMR spectra were proton-decoupled using a gated bi-level mode. ^13^C NMR spectra were obtained (200 FIDs, 3 min acquisition time) from a 66° radiofrequency pulse repeated every second (25,000 Hz spectral width). Lorentzian line broadening of 15 Hz was applied before Fourier transformation for both ^31^P and ^13^C NMR spectra. Assignments of chemical shifts and the metabolite quantitation method were described elsewhere [8-10,30].

Only livers maintaining a total ATP/Pi ratio, measured by ^31^P NMR, during the first 10 minutes of perfusion were kept for entire protocol (total perfusion duration: about 30 min).

### Determination of mitochondrial ATP synthesis

At each time, the whole liver total ATP content reflects a dynamic equilibrium between ATP synthesis and consumption. In this condition, suppression of cytosolic and mitochondrial ATP production makes it possible to calculate the *in situ *rate (R) of mitochondrial ATP turnover [21]. For this purpose, perfusion was performed by first using iodacetate (IAA, 0.5 mmol/L, 2 min) to inhibit the couple glyceraldehyde 3-phosphate dehydrogenase/phosphoglycerate kinase (glycolysis inhibition). After 5 min to reach a new steady state of ATP content (without glycolytic ATP contribution), KCN (2.5 mmol/L, 10 min) was added to inhibit cytochrome oxidase (oxidative phosphorylation) leading to a fall of ATP content.

Since some authors have suspected non specific effects of IAA [18], we have previously performed a work of validation demonstrating that used in above conditions of both concentration and delay, IAA had no significant effect on other pathway than glycolysis [9]; in particular, we had measured only a transient (5 min) VO_2 _increase (<10 ± 2%) which returned to baseline. We have also validated KCN use by kinetic studies of liver ATP content in presence of carboxyatractyloside [9] demonstrating that in response to the large KCN-induced decrease of proton motive force, liver cytosolic ATP was not hydrolyzed *in situ *by mitochondria.

Calculation of mitochondrial ATP turnover: after KCN addition, the mitochondrial ATP content fell according to the following equation: ATP = A.exp^-kt^, (A, expressed in μmol.g^-1 ^liver wet weight, being the ATP content at the time of KCN addition). The mitochondrial ATP turnover was R(t_0_) = -A.k (with k expressed in min^-1 ^and R expressed in μmol. min^-1^.g^-1 ^liver wet weight) since ATP synthesis Rate = ATP consumption Rate at the onset of KCN addition (t_0_).

### Statistics

All results were expressed as means ± SEM. Statistical analysis was performed using one-way analysis of variance (ANOVA) for all data analysis. A post-hoc t-test was performed following the analysis of variance (P value lower than 0.05 was considered to be significant).

## Results

### Change in liver glycogen content after feeding

**Figure 2:**
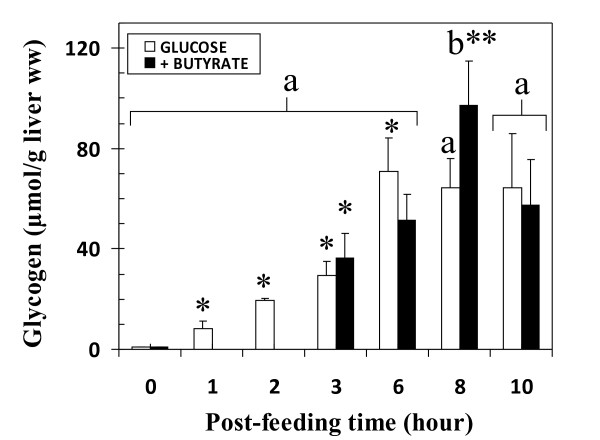
**Kinetic of liver glycogen resynthesis according to type of force-feeding in fasted rats (expressed in glucosyl units)**. m ± SEM. n varied from 6 to 12 for each delay. Isocaloric (7.28 cal) force-feeding (expressed per g body weight) consisted of 18.2 mg glucose or 14 mg glucose + 1.9 mg butyrate. Experimental conditions reflect the *in vivo *situation at each time of liver excision. Labeled means at a time without a common letter differ (t-test), P = 0.05. *Different from previous time in the Glucose group and in the Butyrate group, P < 0.01. **Different from 6^th ^hour in Glucose+Butyrate group, P = 0.035.

**Figure 3:**
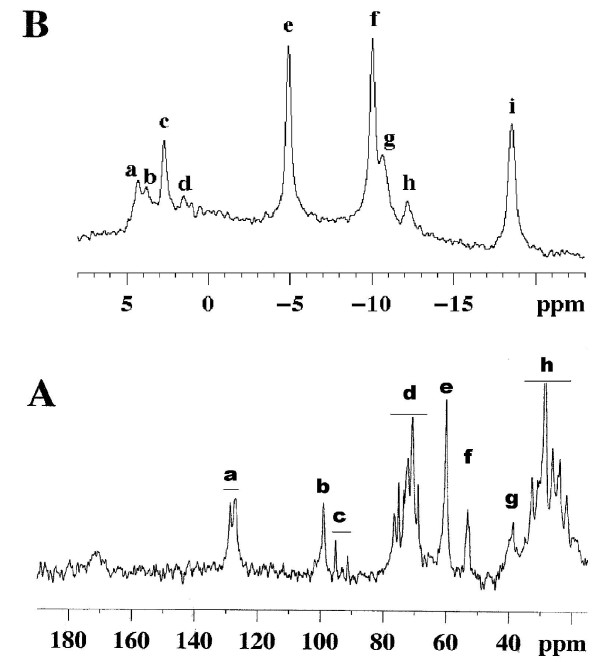
**Typical NMR spectra of perfused and isolated liver of rat fed *ad libitum***. **3A: ^13^C NMR spectrum**. An external silicone reference gives a resonance at 0 ppm. Peak assignments: (a and h) fatty acids chains; (b) C-1 glycogen; (c) C-1α and C-1β glucose (mainly exogenous glucose of the perfusate); (d) glucose and glycogen (C-3β, C-5β glucose, glycogen; C-2 glucose; C-3α glucose; C-2, C-5α glucose, C-5 glycogen; C-4αβ glucose, glycogen); (e) C-6 glucose, glycogen; (f) choline; (g) ethanolamine. The chemical shift scale δ is given in parts per million (ppm) according to: chemical shift (Hz) = δ(ppm) × A(MHz), A being the frequency of the spectrometer. The unit ppm is used owing to the order value (10^-6^) of a constant characterizing the chemical nature of the nucleus. This scale allows easy comparison between spectra obtained in spectrometers operating at different magnetic fields. **3B. ^31^P NMR spectrum**. Major resonances are assigned to (a) phosphomonoesters, (b) phosphocholine, (c) intracellular inorganic phosphate, (d) glycerol-3- phosphorylcholine and glycerol-3-phosphorylethanolamine, (e) nucleoside-'-triphosphates (γNTP) and diphosphates (βNDP), (f) α-NTP and β-NDP, (g, h) nicotinamide adenine dinucleotide and uridine-'-diphosphoglucose, (i) β-NTP.

After 48 hr starvation, the liver glycogen content was undetectable with NMR (Figure 2) compared to a liver isolated from a control rat fed *ad libitum *(Figure 3A). In fasting animals, the glycogen content determined by biochemical assay was 0.85 ± 0.34 μmol.g^-1 ^liver ww [33]. From force-feeding, glycogen increased regularly in a linear time-dependent manner (11.10 ± 0.60 μmol.h^-1^.g^-1 ^liver ww, R^2 ^= 0.95) until the 6^th ^hr post-feeding for the glucose group (Figure 2), and the 8^th ^hr in the butyrate group. Glycogen NMR measurements were validated by comparing them with *in vitro *enzymatic measurements on the liver extracts for each time after force-feeding; glycogen content and time evolution were not different between the two methods [33].

A net glycogenolysis was observed from the 6^th ^hr when animals were fed with glucose alone (-1.60 ± 1.00 μmol.h^-1^.g^-1 ^liver ww, R^2 ^= 0.76, n = 10) and from the 8^th ^hr in the butyrate group (-19.80 ± 2.40 μmol.h^-1^.g^-1 ^ww). At the 10^th ^hr (n = 9) the glycogen content reached a level similar to that in isocaloric glucose force-feeding.

### Correlation between total ATP and glycogen contents during the glycogen synthesis period

**Figure 4:**
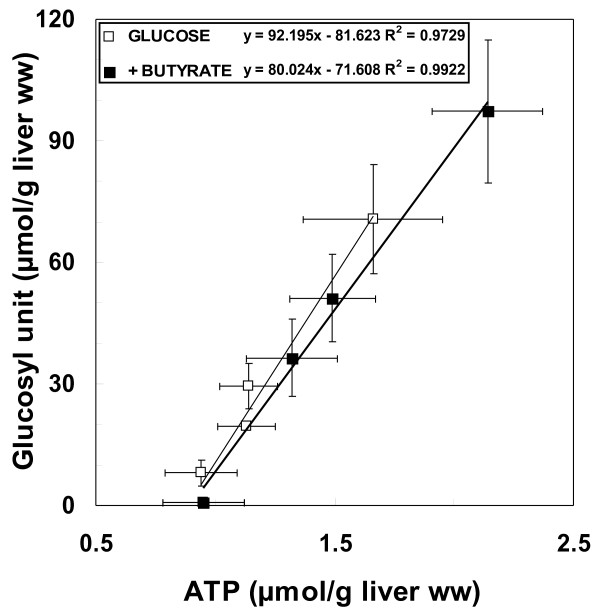
**Correlation between total ATP and glycogen contents during linear glycogen resynthesis**. This linear step lasted until 6 hr and 8 hr in G and G+B groups, respectively. For each time post force-feeding, the glycogen content is expressed according to the total ATP content, the lowest values correspond to the shortest time and the highest values to the longest time. m ± SEM. n varied from 6 to 12 for each delay. Isocaloric (7.28 cal) force-feeding (expressed per g body weight) consisted of 18.2 mg glucose or 14 mg glucose + 1.9 mg butyrate.

A typical ^31^P NMR spectrum of liver fed rat is shown in Figure 3B. NMR depicted mainly nuclei of the active biochemical forms of molecules. Liver total ATP content in *ad libitum *fed rats was 2.42 ± 0.32 μmol.g^-1 ^liver ww in entire perfused organ, corresponding to 2.70 ± 0.30 μmol.g^-1 ^liver ww when measured by ^31^P NMR on perchloric extracts from freeze-clamped perfused livers (n = 15), due to the demonstrated NMR-visibility of βATP near 90% [34]. After 48 hr starvation, the total ATP content measured in minutes following the force-feeding (time 0) was dramatically decreased (averaging 0.95 ± 0.29 μmol.g^-1 ^liver ww, n = 5 in each group), without increase in NMR-visible Pi resonance. This value, also measured on extracts, was in agreement with the known decrease in hepatic ATP content in rats starved during a minimum of 24 hr [35-38]. After force-feeding, total ATP liver content increased regularly (at a rate of 0.13 ± 0.01 μmol.h^-1^.g^-1 ^liver ww, R^2 ^= 0.92) until the 6^th ^hr in the glucose group and until the 8^th ^hour in the butyrate group. A positive and linear correlation between total ATP and glycogen contents was evidenced only during net glycogen synthesis (until 6 hr and 8 hr for glucose and butyrate groups, respectively). The net production of one μmol liver ATP (cytosolic and mitochondrial) was concomitant with a net glycogen synthesis of 92 and 80 μmol glucosyl units in the glucose and butyrate groups, respectively (Figure 4).

### Mitochondrial ATP production after force-feeding

In 48-hr starved rats, and immediately after force-feeding (0 hour), the rate of mitochondrial ATP synthesis (averaging 0.47 ± 0.12 μmol.min^-1^.g^-1 ^liver ww (n = 3 in each group)), tended to be lower than in *ad libitum *fed rats: 0.92 ± 0.22 μmol.min^-1^.g^-1 ^liver ww, n = 3). This is in agreement with a decrease in the content of the ATP synthase complex observed in liver of 18-hr starved rats [39].

**Figure 5:**
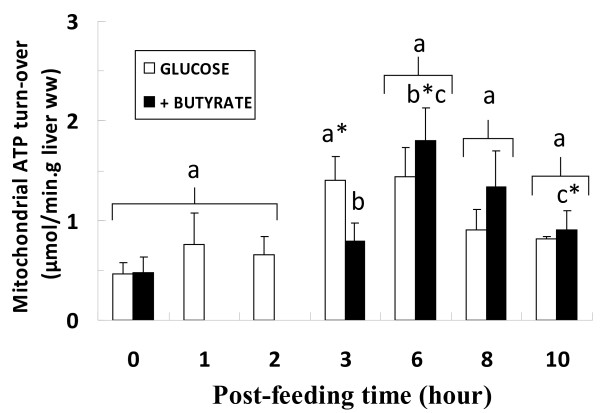
**Effect of presence of butyrate in force-feeding bolus on time-course of mitochondrial ATP synthesis rate**. m ± SEM. n varied from 6 to 12 for each delay. Isocaloric (7.28 cal) force-feeding (expressed per g body weight) consisted of 18.2 mg glucose or 14 mg glucose + 1.9 mg butyrate. Experimental conditions reflect the *in vivo *situation at each time of liver excision. Labeled means at a time without a common letter differ (t-test), P = 0.05. *Different from previous time within each group ≤0.05. **Different from 6^th ^hour in Butyrate group, P < 0.04.

After force-feeding with glucose alone, mitochondrial ATP turnover remained stable during the first 2 hr and then rapidly increased about two-fold at the 3^rd ^hr (n = 10, P = 0.05 *vs *2^nd ^hr), the highest value being reached at the 6^th ^hr. It decreased after the 6^th ^hr to reach the basal level at the 10^th ^hr (n = 9) (Figure 5).

In the butyrate force-fed group, mitochondrial ATP turnover remained stable during the first 3 hr (0.79 ± 0.18 μmol.min^-1^.g^-1 ^liver ww, n = 12), a value significantly lower than in the glucose group (n = 10) (P = 0.05). Then ATP turnover increased to 1.80 ± 0.33 μmol.min^-1^.g^-1 ^liver ww at the 6^th ^hr post force-feeding (n = 9, P = 0.01 *vs *3^rd ^hr). After that, turnover decreased until the 10^th ^hr to a level similar to that observed in the glucose group (n = 9, P < 0.04 *vs *6^th ^hr).

The time constant (k) globally mimicked the changes in ATP turnover from the value of 0.53 ± 0.07 min^-1 ^(n = 3) in starved rat at 0 hr feeding. At the 3^rd ^hr, k was 1.55 ± 0.21 and 0.96 ± 0.19 min^-1 ^in glucose (n = 10, P = 0.01 *vs *0 hr) and glucose+butyrate (n = 12) groups, respectively. The maximal value in the butyrate group was obtained at the 6^th ^hr (1.43 ± 0.31 min^-1^, n = 9, P = 0.05 *vs *0 hour). At the 10^th ^hr, k was 0.68 ± 0.08 and 0.51 ± 0.26 min^-1 ^in glucose (n = 9) and glucose+butyrate (n = 9) groups, respectively.

## Discussion

Gut microbial activity can lead to the production of SCFAs such as butyrate. A hepatic butyrate removal close to 100% has been evidenced in rats and human [4,5]. This raises the question of its effect in this central organ which is highly involved in carbohydrate metabolism.

Our aim was to simultaneously monitor by NMR spectroscopy in the whole rat liver (i) total ATP content and its unidirectional flux of mitochondrial synthesis and (ii) glycogen repletion, at different times following the force-feeding of rats with glucose alone or glucose plus butyrate.

In our experimental nutritional conditions after glucose uptake by the liver, glucose oxidation produced free energy, some being directly available for cellular activity and the rest for ATP storage. ATP is produced by glucose oxidation in two ways. First, it can be directly synthesized in cytosol *via *glycolysis, its end product, pyruvate, entering in the TCA cycle. Second, the reduced cofactors (NADH+H^+^, FADH_2_) resulting from the latter oxidative pathways are re-oxidized in mitochondria by the respiratory chain, ATP synthesis occurring by coupling between respiration and phosphorylative activity. Besides its oxidation, glucose leads to glycogen storage *via *glycogen synthesis. In this study, the liver glycogen content measured *ex vivo *reflected the *in vivo *balance between both glycogen synthesis and glycogen consumption. A net glycogen synthesis was observed 30 min after glucose bolus (data not shown), suggesting the rapid bioavailability of glucose. The glycogen storage in glycogen-depleted liver was linear during the first 6 hr after glucose-feeding. Total ATP content increased concomitantly with the progressive repletion of hepatic glycogen, thus suggesting a relationship between glycogen storage and a change in hepatic ATP content. The linear correlation between total ATP and glycogen contents was evidenced only during the step of net glycogen synthesis; the net production of one μmol liver ATP (cytosolic and mitochondrial) was concomitant with a net glycogen synthesis of 80-90 μmol glucosyl units. Glycogen synthesis needs UTP and then ATP, which must be supplied concomitantly. Since the cellular ATP content reflects the balance between the unidirectional fluxes of production and consumption at any given time, any increase in ATP content implies an increase in the rate of unidirectional fluxes of ATP synthesis. To our knowledge, this study is the first to monitor the evolution of the unidirectional flux of mitochondrial ATP supply in the whole liver after glucose feeding.

Although glycogen synthesis requires energy, the total ATP content increased concomitantly with the glycogen content whereas the mitochondrial ATP supply remained stable during the first 2 hr. One ATP was needed to phosphorylate the exogenous glucose, and one UTP (then one ATP) was used to condense one glucosyl unit arising from glucose-1-phosphate. The rate of net glycogen synthesis averaged 11 μmol.hr^-1^.g^-1 ^liver ww theoretically requiring 22 μmol.hr^-1^.g^-1 ^corresponding to 50% of the measured basal flux of mitochondrial ATP production (42 μmol.hr^-1^.g^-1 ^liver ww), the other 50% part contributing to maintain the basal cellular activities namely ionic homeostasis [40,41] and micro tubular migration [41]. In our conditions of totally depleted glycogen the linear increase in total ATP content thus implies another source that can be only the glycolytic pathway from exogenous glucose. The direct evidence of net glycogen synthesis and indirect demonstration of glycolysis lead to postulate for the establishment of a glucose cycling, this cycling being previously described [12,15].

During the initial phase of glycogen repletion, the stored polymer content was likely not high enough to contribute to glycogenolysis or subsequent cycling of glycogen. Any cycling of glycogen [42-44] could predominate only when the ATP furniture is sufficient and when the hepatic glycogen store becomes sufficient (after 2 hr). This is in agreement with the previous described absence of indirect glycogen synthesis for 2 hours after glucose feeding in rat [44]. Since the net rate of glycogen synthesis was constant, any onset of unidirectional glycogenolysis flux must be algebraically compensated by an increase in the unidirectional flux of glycogen synthesis with the same magnitude. Since an increase in this synthesis flux needs an increase in UTP supply, the increase in the ATP turnover observed at the 3^rd ^hour could be related to this process. This hypothesis of late glycogen cycling is supported by the evidence of progressive glycogenolysis during *ex vivo *incorporation of enriched glucose in newly synthesized glycogen in the isolated perfused liver of fasting rats [45]. Thereafter, a decrease in the unidirectional flux of glycogen synthesis is evidenced by the plateau (and the subsequent decrease) of glycogen content observed after 6 hr post-feeding, and may be related in part to the decreased bioavailability of the exogenous glucose. The progressive decrease in mitochondrial ATP turnover could be partly due to the decrease in energy need related to the reduction in unidirectional flux of glycogen synthesis.

Although the linear correlation between total ATP and glycogen contents was not affected by ingested butyrate, the step of net glycogen synthesis and total ATP synthesis was extended from the 6^th ^to the 8^th ^hour post-feeding. Consequently butyrate might delay the establishment of glycogenolysis as it is an energy substrate. In a previous NMR study using ^13^C-glucose enrichment on *ex vivo *perfused liver isolated from fasted rats, we demonstrated a decrease in the rate of glycogenolysis flux in the butyrate+glucose perfused group compared to an isocaloric perfusion of glucose alone [46]. Partial and direct inhibition of phosphorylase activity by butyrate cannot be excluded but remains to be demonstrated. This result reinforces the hypothesis that butyrate may delay the establishment of glycogenolysis.

In another study [33], we showed that one of the hepatic effects of butyrate *in vivo*, as other FFA, is glucose-sparing, which is due to a preferential butyrate oxidation concomitant with glycolysis slow-down [47], promoting glucose disposal in the glycogen synthesis pathway during the post-prandial state. Unlike glucose oxidation, butyrate oxidation leads *via *mitochondrial -oxidation to the production of acetyl CoA. Acetyl CoA is then oxidized *via *the TCA cycle and impairs pyruvate oxidation, the end-product of aerobic glycolysis. The rate of glycolysis and its cytosolic ATP production are thus reduced.

For the first time, we demonstrate that butyrate ingestion delays from the third hour to 6^th ^hour the increase in the unidirectional flux of mitochondrial ATP supply. Since butyrate oxidation is known to increase liver respiration [8], a stimulation of mitochondrial ATP production could be expected rapidly after bolus feeding. However, a controlling and inhibiting effect of butyrate *per se *was previously described in the isolated perfused rat liver, leading to reduced mitochondrial phosphorylation activity and a large decrease in oxidative phosphorylation yield [9]. As long as butyrate bioavailability and its oxidation were maintained, the onset of glycolysis and the stimulation of the unidirectional flux of mitochondrial ATP supply were delayed.

## Conclusion

The strength of this study is that it is the first to measure simultaneously the hepatic changes of glycogen repletion and the rate of mitochondrial ATP synthesis after feeding of 48 hr starved rats in an experimental condition reflecting *in vivo *changes. The main results are the evidence of (i) a linear relation between total ATP and glycogen contents only during the net glycogen synthesis, (ii) a late and transient increase in rate of mitochondrial ATP supply probably linked to glycogen metabolism and (iii) that butyrate delayed this late and transient increase of ATP turnover. Whether these phenomena are dependent on insulin remains to be investigated.

## Abbreviations used

ATP: adenosine triphosphate; FFA: free fatty acids; FF: force-feeding; KHB: Krebs-Henseleit buffer; k: time constant; NMR: nuclear magnetic resonance; R: ATP turnover; SCFA: short-chain free fatty acids; ww: wet weight.

## Competing interests

The authors declare that they have no competing interests.

## Authors' contributions to manuscript

JLG designed and conducted the research, analyzed the data and wrote the paper, HR performed the experiments and analyzed the data, HG wrote the paper, MCB conducted the research and wrote the paper. All authors read and approved the final manuscript.
